# Suppression and reversal of motion perception around the time of the saccade

**DOI:** 10.3389/fnsys.2015.00143

**Published:** 2015-10-31

**Authors:** Adam Frost, Matthias Niemeier

**Affiliations:** Department of Psychology, University of Toronto at ScarboroughToronto, ON, Canada

**Keywords:** saccades, perisaccadic suppression, motion, V5

## Abstract

We make fast, “saccadic” eye movements to capture finely resolved foveal snapshots of the world but these saccades cause motion artefacts. The artefacts go unnoticed, perhaps because the brain suppresses them through subcortical oculomotor signals feeding back into visual cortex. Opposing views, however, claim that passive mechanisms suffice: saccadic shearing forces might render the retina insensitive to the artefacts or post-saccadic snapshots might mask them before they enter consciousness. Crucially, only active suppression could explain perceptual changes that precede saccades but existing evidence for presaccadic misperception are ill-suited for addressing this issue: Previous studies have found misperceptions of space for objects briefly flashed before saccades, but perhaps only because observers confused the timing of flashes and saccades before they could be tested (“postdiction”), and presaccadic motion perception might have appeared to decline because motion stimuli persisted past eye movement onset. Here we addressed these concerns using briefly flashed two-frame animations (50 ms) to probe people’s motion sensitivity during and around saccades. We found that sensitivity declined before saccade onset, even when the probe appeared entirely outside the saccade, and this sensitivity decline was present for motion in every direction relative to saccade, ruling out problems with postdiction. Intriguingly, brief periods during the saccade produced negative sensitivity as if motion was reversed, arguably due to postsaccadic enhancement. These data suggest that motion perception is minimized during saccades through active suppression, complementing neurophysiological findings of colliculo-pulvinar projections that suppress the cortical middle temporal area around the time of the saccade.

## Introduction

Objects quickly crossing the visual field are distracting. Imagine yourself gazing out the window of an apartment that a thoughtless architect has squeezed against the subway tracks of a North American city. Train after train rushes by just beyond the glass and startles you causing disorientation and nausea. Similarly sickening could be the visual blur that your eyes cause on their retinas three times per second when they make quick “saccadic” eye movements.

Fortunately however, conscious perception remains undisturbed by the saccadic blur or “grayout” (Mitrani et al., 1970; Campbell and Wurtz, 1978) because sensitivity drops during saccades. For example, flashes of light are poorly perceived (Dodge, 1900), and gratings that should be discernible at saccadic velocities become invisible (Diamond et al., 2000). This saccadic suppression of vision could arise from either active or passive mechanisms.

Passive accounts of perisaccadic suppression of vision are attractive for their parsimony. These accounts argue that vision can be entirely oblivious to the movements of the eye and that no active saccadic suppression is necessary (Castet et al., 2001) because saccadic blur functions as its own mask (Mitrani et al., 1970), because pre- and post-saccadic vision masks the blur of the saccade (Mackay, 1970; Matin et al., 1972; Campbell and Wurtz, 1978; Judge et al., 1980), and because shearing forces of the rotating eyes blind them transiently (Richards, 1969; Castet and Masson, 2000; Castet et al., 2001).

Active suppression mechanisms would be more complicated because they would require extraretinal signals about eye movements. However, there is neurophysiological evidence that such extraretinal signals are subserved by neural projections that have been found to originate from oculomotor signals. One such projection passes through the lateral geniculate nucleus (LGN) of the thalamus where it inhibits (and, postsaccadically, facilitates) neural activity (e.g., Lee and Malpeli, 1998; Reppas et al., 2002), especially within the magnocellular system (Ramcharan et al., 2001). Another pathway projects from the superior colliculus to the thalamic pulvinar to then inhibit motion area MT some 60 ms before saccade onset (Berman and Wurtz, 2011). This is consistent with changes in activity in MSTd and other cortical areas of the dorsal stream starting several tens of milliseconds before saccade onset (e.g., Thiele et al., 2002; Ibbotson et al., 2008; Bremmer et al., 2009; for possibly presaccadic modulations in striate and extra-striate cortex: Duffy and Lombroso, 1968; Kleiser et al., 2004; Sylvester et al., 2005; but see, e.g., Thilo et al., 2004).

Crucially, the physiological evidence for presaccadic suppression of dorsal areas and thus arguably of spatial and of motion information is complemented by perceptual data suggesting that people experience visual misperceptions that begin before the start of saccades. One line of research probed perception with individual stimuli briefly flashed at different times around saccade onset. Observers were less likely to detect the flashes (e.g., Volkmann et al., 1968) and they seemed to misperceive the locations of presaccadic stimuli either as shifted or compressed towards the saccade target before the eye started to move (e.g., Matin and Pearce, 1965; Honda, 1989, 1993; Cai et al., 1997; Morrone et al., 1997; Ross et al., 1997; Lappe et al., 2000; Kaiser and Lappe, 2004; Kis et al., 2009). Consistent results come from a different approach that tested motion perception directly: Shioiri and Cavanagh (1989) presented observers with a pattern of random dots that was mostly stationary for 2 s. However, at random times during the 2 s the pattern shifted in a single step. This shift was difficult to see during saccades, starting several tens of milliseconds before observers moved their eyes.

No such presaccadic changes in perception would be explicable with passive accounts of perisaccadic suppression of vision and therefore would serve as a strong argument against passive accounts (although also see, e.g., Burr et al., 1994, 1999). However, presaccadic misperceptions could be methodologically compromised. Presaccadic misperception of spatial locations could be due to “postdiction”. That is, given the nature of perceptual experiments participants report their percepts well after a saccade, and the delay might cloud people’s ability to discern the timing of visual stimuli relative to their eye movements. Also, it is not entirely clear how misperceptions of spatial positions would pertain to the physiological data of suppression of motion information (reduced visibility before saccades, Volkmann et al., 1968, could impact motion perception but not if the motion stimuli were clearly above perceptual thresholds).

Shioiri and Cavanagh’s (1989) report of declined motion perception is difficult to reconcile with postdiction. However, Shioiri and Cavanagh (1989) results could be inaccurate in time because the stimuli reached into the saccades (their control experiment 3 blanked the stimulus for 50 ms but that might have created a so-called blanking effect; Deubel et al., 1996). Other studies used briefer motion stimuli but only after saccade onset (Burr et al., 1982, 1999; Ilg and Hoffmann, 1993).

In sum, there is a long-standing debate on whether saccadic suppression of vision is due to active or passive mechanisms with presaccadic changes in perception as the, arguably, strongest argument in favor of active mechanisms. Surprisingly however, to date no unequivocal support for presaccadic suppression of perception exists. Therefore, here we combined the advantages of previous paradigms and presented a brief (2-frame) motion stimulus randomly flashed around the time of saccades. Also, we tested the influence of saccade direction on motion perception to test whether saccadic blur would mask motion perception specifically along the plane of the saccade (Mitrani et al., 1970), and also because previous studies have found direction to matter for some forms of perisaccadic perception (e.g., Niemeier et al., 2003, 2007; Kaiser and Lappe, 2004). We confirm and extend Shioiri and Cavanagh’s (1989) finding of a presaccadic decline of motion perception regardless of motion direction and report a novel phenomenon of reversed motion perception during the saccade. Our data provide important new insights into the active mechanisms of perisaccadic perception of motion.

## Materials and Methods

### Participants

Twenty-four students at UTSC (median age: 19) gave their informed and written consent to participate in this study. Experiment 1 tested 8 students, Experiment 2 tested 16, and Experiment 3 tested 6. The control experiment tested 3 participants. 6 people participated in both Experiments 1 and 2, and one person (author AF) participated in Experiment 1 and the control experiment. All participants were healthy with normal or corrected-to-normal vision. Procedures were approved by the Human Participants Review Sub-Committee of the University of Toronto and have therefore been performed in accordance with the ethical standards laid down in the 1964 Declaration of Helsinki.

### Apparatus and Procedures

Participants sat in a dimly lit room with their head stabilized by a chin rest. Thirty centimetres in front of them at eye level, a 21^″^ CRT monitor (Sun Microsystems; Resolution: 1024 × 768; Refresh rate: 100 Hz) presented stimuli that were generated by programs written for MATLAB with the Psychophysics and EyeLink toolbox extensions (Brainard, 1997; Pelli, 1997; Cornelissen et al., 2002). Eye position was recorded using an Eyelink 2 system (SR Research, 500 Hz), and participant responses were collected using a numeric keypad.

**Figure 1:**
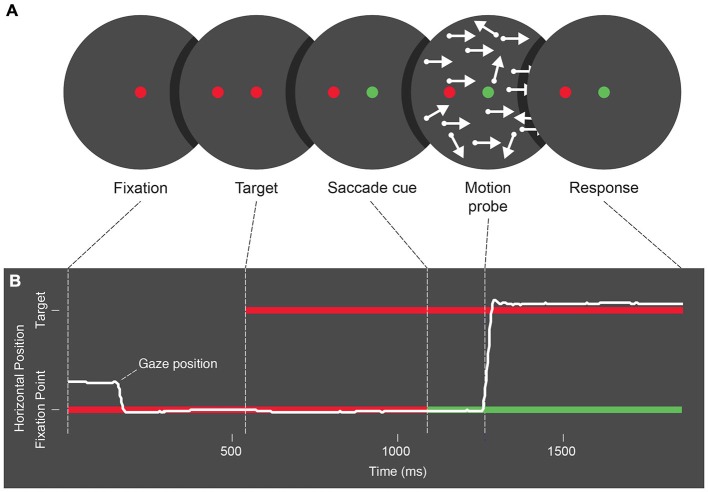
**(A)** Schematic of the stimulus presentation sequence used across Experiments 1, 2 and 3. Trials begin with a fixation period, followed by the appearance of a saccade target. Next the color of the fixation point changes as a cue for participants to make an eye movement, and following the cue a two-frame random dot motion probe is presented. After refixation following the eye movement, participants give a 2 alternative forced choice response to the motion probe. **(B)** Schematic representation of eye position during each trial phase. Participants begin by fixating, and following the saccade cue, an eye movement is made towards the saccade target.

As illustrated in Figure 1, trials first showed a red circle (0.6° across) on a black (luminance: 0.1 cd/m^2^) background, and participants were asked to fixate it. Five hundred to 1000 ms later, a similar target circle appeared 7.2° away. Both circles appeared on opposite sides of the screen center along the horizontal or vertical meridians and at a distance of 1.8° and 5.4°, respectively. Another 500–1000 ms later the fixation point turned green to signal participants to move their eyes. At targeted times before, during, or after saccade onset (see below), a brief two-frame motion probe (25 ms/frame), was flashed. Trials concluded with a button-press response and return to fixation.

#### Motion Probe

The motion probe consisted of white dots (luminance: 18.8 cd/m^2^, diameter: 0.35°), placed at random across the screen. Pilot testing confirmed that the dots were clearly visible during eye movements, that is, we found that the presence or absence of dots was correctly reported 99% of the time when triggered by and flashed during saccades, and 98% of time when flashed outside of saccades (with all errors likely due to button confusion). In total there were 5000 dots, but only ~1700 dots were actually shown. We hid any dots positioned or moving more than 22.5° away from screen center to create a circular aperture, and to minimize visual distraction we deleted dots within 2.9° wide bar-shaped zones along the screen’s horizontal and vertical meridians. In the transition from frame 1 to frame 2 (dispersed across 5 refresh cycles of the monitor with the third cycle dithering the two frames), all dots traveled 0.9°, where 80% of them moved coherently in one of the four cardinal directions, and 20% moved in random directions. We found that 80% coherence was easily perceived without eye movements (>85% correct test trials) by experienced participants. Novice participants recruited, in part, for Experiments 2 and 3 required a brief training of 40–320 trials (as needed) during which coherence was gradually lowered from 95% down to 80%. Experiment 1 and 2 used a two-alternative forced-choice approach to test motion either parallel or orthogonal to the saccade in separate blocks. Experiment 3 compared forward and backward motion relative to the saccade and therefore asked participants to detect coherent motion among trials with incoherent motion.

#### Motion Probe Timing

Motion onset (defined as the time when the motion probe’s second frame appeared) deliberately targeted certain perisaccadic times. Specifically, Experiment 1 analyzed participants’ saccades online and updated, trial-by-trial, average individual saccade latencies to “guess” future latencies and present the motion probe accordingly. Experiments 2 and 3 also guessed latencies but aimed to present the motion probe during a “before saccade” epoch of ~100 ms prior to saccade onset for one third of the trials. A second third of trials targeted a “during” epoch, that is, they flashed probes as soon as the actual saccade was detected (eye velocity > 30 deg/s, delay: ~9.5 ms). The last third of trials targeted an “after” epoch 100 ms after detected saccade onset. Trials were sorted *post hoc* according to when the probe actually appeared relative to saccade onset.

#### Control Experiment

To confirm that our motion probes actually tested motion perception we used a motion adaptation paradigm (Blake and Hiris, 1993). Participants fixated on a small (0.6° across) circle at the center of the screen and watched 60 s of 100% coherent leftward or rightward random dot motion, and each trial added another 10 s of top-up adaptation. After a random 125 ms to 1210 ms post-adaptation delay, participants viewed 2-frame probes of horizontal motion and responded indicating the probe’s coherent motion direction with a two-alternative forced-choice response. The proportion of coherently moving points in the probe was randomly selected from trial to trial at increments ranging from 50% in the adaptation-congruent direction to 50% anti-adaptation.

### Data Analysis

For Experiments 1 and 2 we separated trials according to whether motion ran parallel or orthogonal to the saccade, and for Experiment 3 we separated trials with forward and backward motion. For each of these motion categories, we collected trials into temporal bins depending on the timing of the motion probe (i.e., the time when the motion probe’s second frame appeared) relative to saccade onset. For Experiment 1, with its wide range of tested times, we created bins of 50 trials from each participant’s 1447–2579 individual trials, with the first bin starting at saccade onset. That is, we collected a participant’s first 50 trials that had presented motion probes upon or right after saccade onset and we set the time of that bin to the average of all included trials. Then we collected the next 50 trials after saccade onset into the second bin, and we continued creating bins until all postsaccadic trials were binned. Equivalently, we pooled all presaccadic trials into bins of 50. Bins with less than 50 trials at the fringes of the tested range were lumped together with neighboring bins, and bins with no false alarms were combined with nearby bins or otherwise eliminated to avoid producing d’ values of infinity. Also, for more specialized questions we recycled trials to create temporal bins outside, immediately before, and during saccades (see “Results” section). Similar bins were also used for Experiments 2 and 3 where we inspected motion probes presented outside the saccade or right after saccade onset.

**Figure 2:**
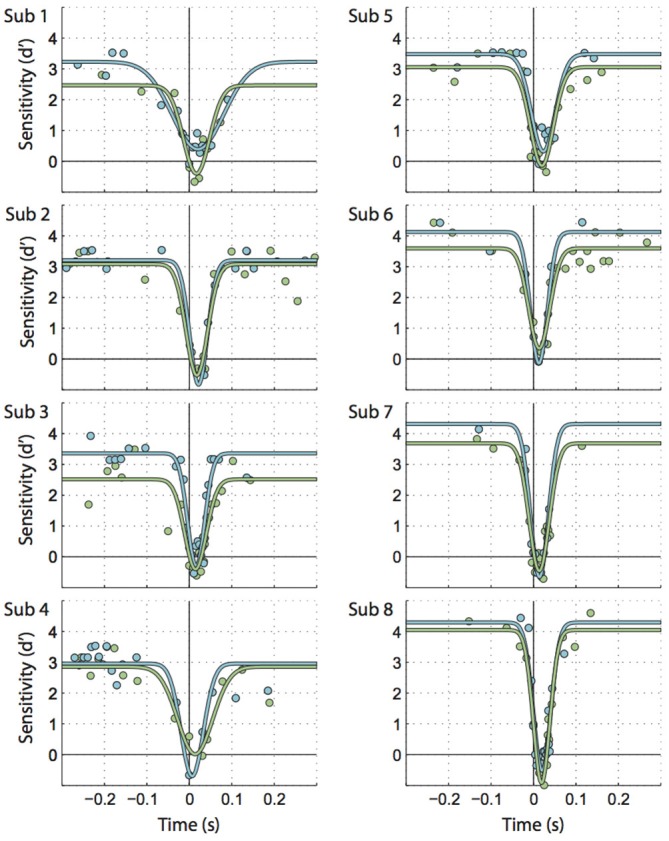
**Individual participants’ data illustrating the time course for saccadic motion suppression.** Times along the X axis are the difference between motion onset time and saccade onset. Circles represent bins of trials, fitted by inverted Gaussian curves. Each graph represents 1066 trials on average. Green: motion parallel to the saccade, blue: motion orthogonal to the saccade.

Then, for each bin we calculated sensitivity (d^’^, i.e., z-transformed hit rates minus z-transformed false alarm rates, e.g., Figure 2) to remove influences of response or perceptual biases (e.g., biases caused by retinal smear). In Experiments 1 and 2 we converted two-alternative forced-choice responses for parallel motion such that we coded trials as “hits” and “misses” where participants saw forward motion and reported it correctly or incorrectly, respectively, and we coded trials as “correct rejections” and “false alarms” where participants were shown backward motion and correctly (or incorrectly) reported it. For orthogonal motion no equivalent general geometric rule exists. Sensitivities as reported here were obtained as follows: for trials with leftward saccades we coded correctly reported downward motion as hits (misses, correct rejections, and false alarms were coded equivalently), for trials with rightward saccades, upward motion counted as hits, for trials with downward saccades leftward motion counted as hits, and for trials with upward saccades rightward motion counted as hits. We tried other strategies of pooling trials and calculating d^′^ values but found that these strategies yielded very similar results. Also, for Experiment 3 (where participants were asked to detect motion, rather than choose between two kinds of motion) trials with forward and backward motion relative to saccades were used to create separate counts of hits and misses. Trials with incoherent motion were *a priori* assigned to the forward and backward motion categories and thus served as separate counts of participants’ correct rejections and false alarms so as to attain statistical independence.

**Figure 3:**
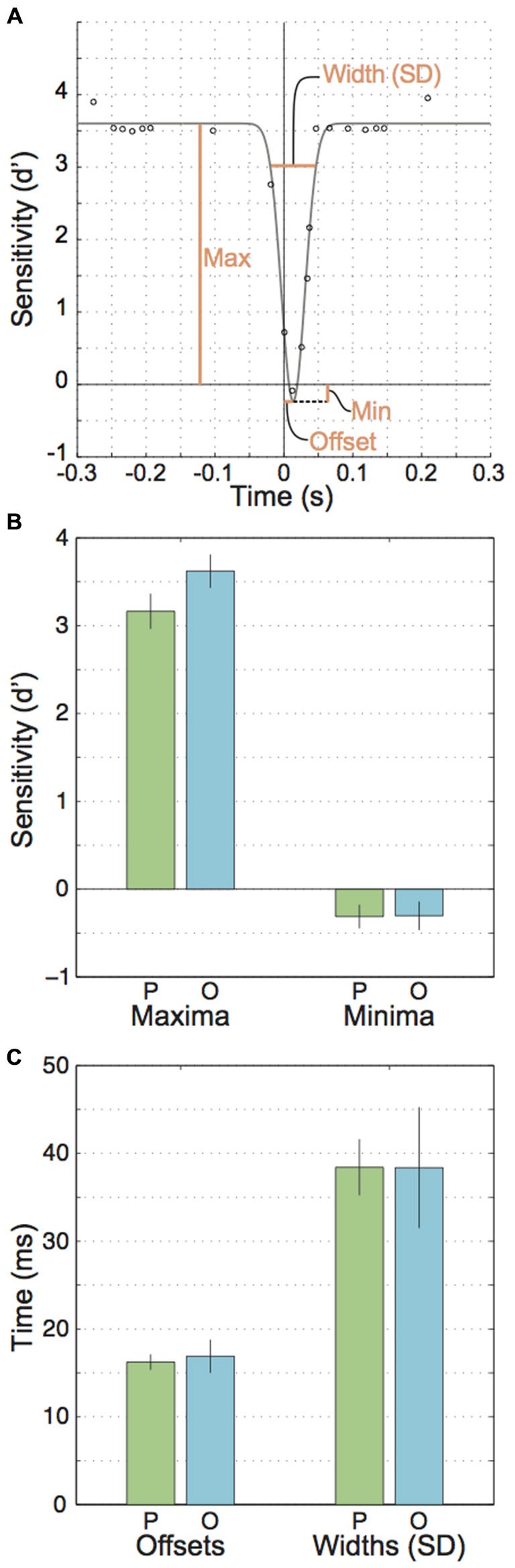
**Parameters for the fitted Gaussian curves across participants. (A)** An illustration of the free parameters used to fit inverted Gaussians to the data points. **(B)** Mean parallel and orthogonal parameters for the gaussians’ maxima and minima. **(C)** Mean parallel and orthogonal parameters for the fitted Gaussian’s offsets from zero and widths (SDs).

Next, we submitted sensitivity values in the three experiments to different statistical tests of inference. For Experiment 1 this included one additional step where we fitted inverted Gaussian curves with parameters maximum, amplitude, standard deviation and offset (Figure 3A) to the data. The best fits for these parameters were then further inspected using *t*-tests.

## Results

### Experiment 1

Figure 2 shows individual sensitivities to motion parallel and orthogonal to the saccade as a function of time relative to saccade onset (green and blue circles). Inverted Gaussians fitted to these data (also see Figure 3A) reflected the well-known perisaccadic decline in motion sensitivity (Shioiri and Cavanagh, 1989), i.e., the amplitudes of the fitted Gaussians were significantly larger than zero (parallel motion: *t*_(7)_ = 13.17, *p* < 0.001, *M* = 3.48; orthogonal motion: *t*_(7)_ = 14.87, *p* < 0.001, *M* = 3.92).

Next, we looked at extra- vs. peri- and intrasaccadic perception separately. Extrasaccadic sensitivities (i.e., the maxima of the inverted Gaussians, Figures 3A,B, left) were 3.2 and 3.6 for parallel and orthogonal motion, respectively, reflecting that the motion probe was easily perceived during fixation. Nevertheless, motion perception during fixation was influenced by the upcoming or recent saccade in that motion orthogonal to saccades was better perceived than parallel motion (*t*_(7)_ = 4.57, *p* = 0.003), likely due to more sustained effects of attention directed to the saccade target rather than perisaccadic suppression (see “Discussion” section).

In contrast, intra- and perisaccadic perception showed no or subtle influences of motion planes. Intrasaccadic sensitivity reached a minimum (Figure 3A) that was very similar for parallel and orthogonal motion (*t*_(7)_ = 0.049, *p* = 0.96) and close to zero (parallel: *t*_(7)_ = 2.31, *p* = 0.054, *M* = −0.31; orthogonal: *t*_(7)_ = 1.86, *p* < 0.105, *M* = −0.30), indicating a profound inability to perceive the motion stimulus during saccades, regardless of motion plane. This minimum occurred at about the center of the period where the eye is in motion (parallel offset: 16.2 ms, orthogonal: 16.9 ms, *t*_(7)_ = 0.55, *p* = 0.598, Figure 3C, left). Finally, the standard deviations of the Gaussians for parallel and orthogonal motion were nearly identical (*t*_(7)_ = 0.006, *p* = 0.995, parallel: 38.4 ms, orthogonal: 38.4 ms). Taking offsets and standard deviations together one could conclude that motion suppression occurred at least 21.5 ms before saccade onset. However, this estimate could be misleading because (a) inverted Gaussians might not be accurate enough to describe the exact time line of perisaccadic suppression; and (b) a motion probe presented at 22 ms would partially reach into the saccade and this could confound the estimates of perception before saccade onset.

**Figure 4:**
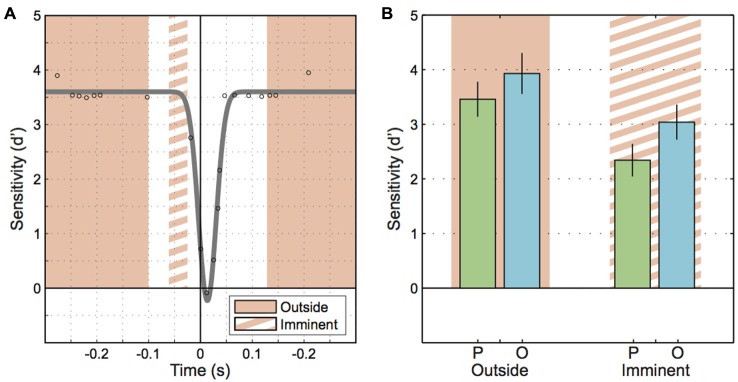
**Motion sensitivity when a saccade is imminent. (A)** Schematic illustrating the epochs for motion onset that fall outside of the perisaccadic interval (solid orange region) and the epoch where saccades are imminent (orange striped region). **(B)** Sensitivity to parallel and orthogonal motion probes outside of the perisaccadic interval and when saccades are imminent. Error bars represent the standard error.

To avoid these problems, we sidestepped the fitted inverted Gaussians and reanalyzed the data focusing on two particular time windows. We defined an “imminent” time window or epoch that included motion probes presented between 65 ms and 25 ms before saccade onset (Figure 4A, orange-stripe region), that is, the window included motion probes that were presented just before saccade onset but excluded probes that reached into the saccade. An “outside” window collected all trials with motion probes presented well before (300 ms to 100 ms) and well after (135 ms to 300 ms) saccade onset (Figure 4A, orange-solid regions). Sensitivity values for these times submitted to a 2-way repeated-measures ANOVA produced a main effect of “Epoch” (imminent/outside; *F*_(1, 7)_ = 16.67, *p* = 0.005; Figure 4B). This shows that motion sensitivity started to decline before saccade onset. Furthermore, the ANOVA yielded a main effect of “Motion plane” (*F*_(1, 7)_ = 9.08, *p* = 0.020), consistent with the extrasaccadic differences between parallel vs. orthogonal sensitivity mentioned earlier (Figure 3B, left). Interestingly, there was no interaction between “Epoch” and “Motion plane” (*F*_(1, 7)_ = 0.58, *p* = 0.471), indicative of two independent mechanisms with additive effects on sensitivity.

**Figure 5:**
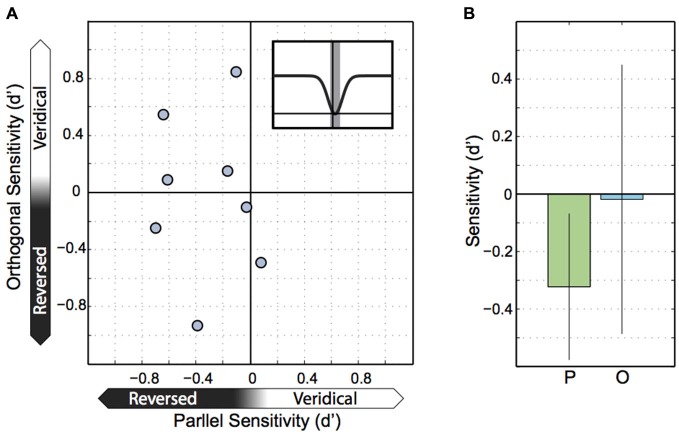
**Sensitivity at peak suppression. (A)** Parallel and orthogonal motion sensitivity at peak suppression. Each point represents parallel and orthogonal motion for one participant, with parallel motion on the X axis and orthogonal motion on the Y axis. **(B)** Sensitivity to parallel and orthogonal motion across participants at peak suppression. Error bars represent the 95% confidence interval.

Our final analysis of the Experiment 1 data was motivated by the observation that several participants exhibited intrasaccadic sensitivities that were negative, rather than zero (Figure 2). Because the inverted Gaussians might have been imperfect descriptions of the true changes in sensitivity and so, might have underestimated sensitivity minima, we resampled trials at participants’ minima. That is, we used the offsets of the inverted Gaussians to estimate when individual people reached their minimum in motion sensitivity and then centered a small time window of ±5 ms on it. In effect, this window included trials with motion probes reaching out of the saccade on both ends. The resulting sensitivities are plotted in Figure 5. Interestingly, negative sensitivity existed for motion running parallel to the saccade in 7 out of 8 participants (*t*_(7)_ = 2.99, *p* = 0.02). These results were clearly not explained by a bias in motion perception to do with the retinal smear from the saccade, instead the sensitivity data indicate that parallel motion probes flashed at the center of the saccade were systematically perceived as reversed. Sensitivity and biases, calculated as d’ and lambda center are independent concepts that can be teased apart mathematically. To illustrate this, we found that forward motion in the same direction as the saccade was correctly reported “forward” with 12% probability, but incorrectly reported as “backward” in the remaining 88% of all cases. In contrast, backward motion was correctly reported as backward 81% of the time and incorrectly as forward 19% of the time. So people were biased due to retinal smear (lambda center = −1.03), but that cannot explain why they reported backward motion more frequently when it was actually forward motion than when it was actually backward motion. Instead the data indicate that perception was affected by a combination of biases and negative sensitivity (d^′^ calculated from these averaged percentages is −0.29, which differs from −0.32 in Figure 5B due to rounding errors). The negative d’ values that we observed suggest that motion in the same direction as the saccade was, trend-wise, reported as motion in the direction opposite to the saccade direction. Also, motion in the opposite direction relative to the saccade was, trend-wise, reported as motion in the same direction. However, for orthogonal motion there was no equivalent systematic trend for sensitivity (*t*_(7)_ = 0.09, *p* = 0.929) (and detailed inspection of the data sets of individual participants for the different saccade and motion directions confirmed that this zero trend was not caused by our approach of calculating sensitivities for orthogonal motion, see “Data Analysis” section). Comparing parallel and orthogonal sensitivities with each other yielded no significant differences (*t*_(7)_ = 1.47, *p* = 0.162; Levene’s test: *F*_(1, 14)_ = 1.64, *p* > 0.05).

### Experiment 2

**Figure 6:**
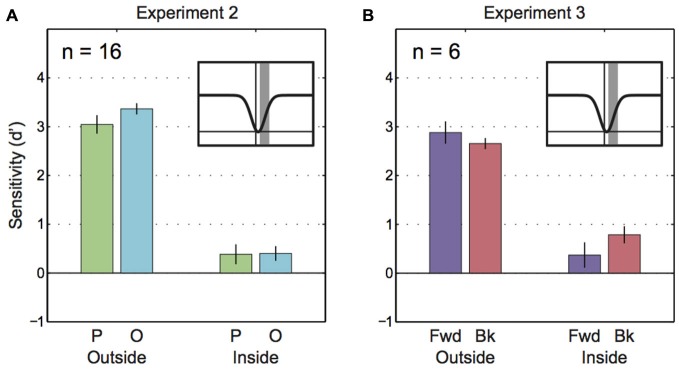
**Saccadic motion suppression in Experiments 2 and 3. (A)** Sensitivity to parallel and orthogonal motion outside of saccades and during saccades for Experiment 2. The inset figure with the gaussian curve shows the time frame for trials that comprise the “inside” epoch (gray bar). **(B)** Sensitivity to parallel and orthogonal motion outside of saccades and during saccades for Experiment 3. The inset figure with the gaussian curve shows the time frame for trials that comprise the “inside” epoch (gray bar).

Because Experiment 1 found that parallel and orthogonal motion perception was largely equal during the saccade (except at the center of the saccades), we followed up with a second experiment that provided a broader sample (*n* = 16 instead of *n* = 8) of participants with greater data density at critical points in time (before, during and after saccade). Also, Experiment 2 included novice participants to rule out the possibility that differences in sensitivity to parallel/orthogonal motion disappear after hours of training in a portion of the tested group. As shown in Figure 6A, we found the expected loss in sensitivity during saccades compared to sensitivity during fixation (main effect “Epoch”: *F*_(1, 15)_ = 180.16, *p* < 0.001). Neither the difference in parallel vs. orthogonal motion sensitivity (main effect “Motion plane”: *F*_(1, 15)_ = 3.11, *p* = 0.10) nor the interaction with epoch were significant (*F*_(1, 15)_ = 0.37, *p* = 0.09). A pre-planned *t*-test indicated that intrasaccadic motion sensitivities along the parallel and orthogonal planes were almost equal (*t*_(15)_ = −0.12, *p* = 0.9). An analysis of minimum sensitivity comparable to the Experiment 1 data (Figure 5) was not possible because motion probes triggered by the saccade appeared later than the probes inspected in Experiment 1. In sum, even with greater statistical power we found no differential effects of motion plane on intrasaccadic sensitivity.

### Experiment 3—Forward vs. Backward Motion

Did parallel and orthogonal motion sensitivities in Experiments 1 and 2 only appear to be similar because parallel data averaged across forward and backward motion relative to the saccade? To test whether saccadic motion suppression selectively targeted backward motion (i.e., the direction of the blur caused by saccades) Experiment 3 looked at sensitivities for forward and backward motion separately (Figure 6B). However, the 2-way ANOVA only revealed a main effect of “Epoch” (*F*_(1, 5)_ = 73.79, *p* < 0.001). There was no statistical influence of “Motion direction” (*F*_(1, 5)_ = 0.37, *p* = 0.57) and the interaction was not significant (*F*_(1, 5)_ = 5.36, *p* = 0.07).

### Control Experiment

**Figure 7:**
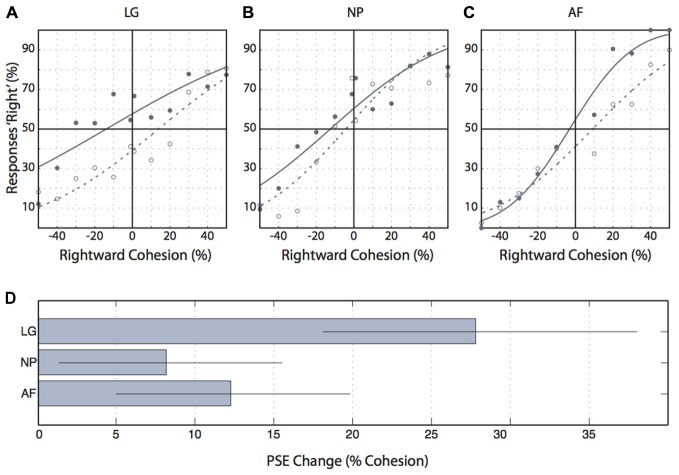
**Control experiment results. (A–C)** Individual participants’ proportions of “rightward” responses for each level of coherent motion. Negative cohesion values indicate leftward coherent motion. Filled circles indicate the proportion of rightward responses following leftward adaptation, and open circles indicate these values following rightward adaptation. Solid lines are cumulative gaussians fitted to the leftward adaptation data and dashed lines are cumulative gaussians fitted to the rightward adaptation data. **(D)** The change in the point of subjective equality (“rightward” responses) from leftward to rightward adaptation. Error bars indicate the 95% confidence interval.

The control experiment presented motion probes during fixation and after adaptation to motion. All three participants showed psychometric functions that were shifted further leftward after rightward adaptation compared to psychometric functions after leftward adaptation (Figures 7A–C). These differences in points of subjective equality superimposed with bootstrapped 95% confidence intervals were significantly different from zero (Figure 7D), consistent with the idea that the 2-frame motion probes employed in our study indeed tested motion perception.

## Discussion

Saccades cause major disruptions in the visual input, yet these disruptions go unnoticed for reasons that are not entirely understood. Active or passive mechanisms of saccadic suppression of motion perception could play a role but only active mechanisms could explain suppression that starts before the eye begins to move. Here we used a short, 2-frame motion probe to accurately map motion perception in time. We found that at least 65 ms to 25 ms before saccade onset perception started to decline, and that about 16.6 ms into the saccade motion perception became profoundly suppressed. Confirming and extending Shioiri and Cavanagh (1989) study on perisaccadic motion perception our data suggest that motion perception is actively suppressed around the time of saccades, and that this suppression is effective and specific.

Saccadic motion suppression must be active because it starts before saccade onset, at a time when the motion probe appears completely outside the saccade. No visual artefacts caused by retinal motion can explain these effects. Also, the effects cannot be due to the postdictive nature of perceptual testing; postdiction could cause people to confuse the onset of visual stimuli relative to saccadic eye movements, but it could not explain confusion about the relative order of our two motion probe frames, with that confusion being specifically aligned with the saccade. In sum, our finding of presaccadic motion suppression complements physiological evidence for extraretinal signals conveyed through tectopulvinar projections and/or projections through the LGN that suppress activity in a range of cortical areas such as areas MT, MST, and other dorsal areas (Ibbotson et al., 2008; Bremmer et al., 2009; Berman and Wurtz, 2011). What is more, our data suggest that the time course of motion suppression onset matches well neurophysiological reports of neural suppression, e.g., of up to 90 ms before saccades in MSTd (see, Ibbotson et al., 2008; Figure 7) and of 60 ms in the pulvinar (Berman and Wurtz, 2011).

This active motion suppression appears to be effective. We found that observers were essentially unable to extract motion information from the probes despite the fact that passive mechanisms were largely absent. That is, the motion probes were so brief that it is unlikely that the probe’s frames masked the transition between them and even so, such masking would be unspecific and affect motion perception outside the saccade the same as inside. Specific masking inside saccades caused by the saccadic blur, however, should affect different motion directions differently but here we found no evidence for direction-dependent suppression despite extensive testing. One exception is that at about the center of the saccade motion sensitivity parallel to the saccade became negative, that is, people perceived forward motion as backward motion and* vice versa*, whereas orthogonal motion perception was not systematically reversed. Saccadic blur, however, could at most reduce motion sensitivity to zero, not reverse it. Instead, we argue that reversed motion perception is a sign for an active mechanism (see below). The only other passive mechanism that remains are shearing forces imposed by the rotating eyes that could render retinal cells dysfunctional during the saccade (Richards, 1969). We cannot rule out these effects but to our knowledge shearing has very limited impact on vision (see, e.g., Ross et al., 2001). We conclude, the substantial decline in motion sensitivity as observed here is largely due to active mechanisms, and thus, active mechanisms constitute a significant, if not the primary, mechanism of saccadic motion suppression in daily life scenarios as well.

One might ask, however, whether our results reflect suppressed motion perception specifically. We argue that this was the case. Our probes must have tested motion perception because perception of probes changed with motion aftereffects. In contrast, the observed decline in sensitivity cannot be explained by suppression of other aspects of perception. It could not be explained by a perisaccadic decline in contrast, because the contrast of the motion probes was clearly above threshold and the probes remained clearly visible during the saccades as we verified in pilot tests (see “Materials and Methods” section). Also, the decline could not be due to poor displacement perception called saccadic suppression of displacement (Bridgeman et al., 1975), because probes were likely too brief to provide useable information to be able to sense displacements or changes in the spatial position of stationary objects, and because saccadic suppression of displacement would be more pronounced parallel to the saccade than orthogonal to it (Niemeier et al., 2003, 2007), whereas our data suggest largely adirectional saccadic suppression.

Nevertheless, we observed two periods during which motion direction mattered. One period outside saccades suggested that people participating in Experiment 1 were better at seeing motion orthogonal to the saccade than parallel to it. We believe that this difference is not directly related to changes in perception around the time of the saccade because the effect occurred across longer stretches of time outside saccades and because the effect did not interact with the perisaccadic decline in sensitivity. Therefore, it is conceivable that this fixational difference comes from less transient, arguably cognitive mechanisms which are beyond the scope of the current study and would require further testing. However, we can speculate that, for example, participants, while preparing for their eye movement, directed their attention to the saccade target and that this attentional bias could have created greater cognitive or perceptual loads along the plane of the planned saccade, thereby limiting people’s ability to perceive or report motion along the same dimension.

A second period of direction-specific motion perception was limited to a small, 10 ms wide time window centerd on people’s individual minimum sensitivities (~16.6 ms after saccade onset) where we found that sensitivity attained negative values but only for motion probes parallel to the saccade. We argue that this difference is independent of the difference during fixation because there is no reason to assume that poorer fixational motion perception could be related to negative sensitivities (note that inverted Gaussians poorly fitted to our data could have created misestimates of intrasaccadic sensitivity along with false differences between parallel and orthogonal motion, but our approach to estimating sensitivities addressed this problem).

The negative sensitivity values imply that people performed significantly below guessing rate, thus, rather than being motion blind they perceived motion as reversed. We believe that the key to understanding this strange effect is the fact that negative sensitivities occurred for probes where the two frames overlapped with the start and end of the saccade, respectively, so that the first frame appeared at a time of substantial motion suppression whereas the second frame coincided with the end of the saccade, so a phase that is known to incur post-saccadic enhancement (Burr et al., 1994). This enhancement is believed to operate independent of saccadic suppression (Ibbotson et al., 2008) and might have a range of influences on perception (Burr et al., 1994). We propose that one such influence is that post-saccadic enhancement might work to the effect of turning motion signals into their opposite, much like a perceptual servomechanism. We speculate that extraretinal signals might cause motion in one direction to trigger a brief burst of activity in neurons with the opposite motion preference. It is interesting to note that such a paradoxical neural response is consistent with Thiele et al.’s (2002) report of a subset of cells in MT and MST that reverse their preferred direction of motion during saccades. Although further neurophysiological tests would be required, reversed motion tuning through extraretinal signals is conceivable because it would be simple to implement, and useful so as to conceal residuals of saccadic blur through active (extraretinally driven) forms of masking. Furthermore, such a mechanism could selectively target motion parallel to the saccade and less so orthogonal motion, given the lesser need to suppress orthogonal motion. That is, it might target orthogonal motion in some people but not in others, consistent with the observed range of positive and negative sensitivities to orthogonal motion.

On a general level, post-saccadic enhancement of perception (Burr et al., 1994) and its neural correlate of increased postsaccadic activity and accelerated spike latencies (e.g., Lee and Malpeli, 1998; Reppas et al., 2002; Ibbotson et al., 2006, 2008) might cause people to perceive time as compressed or reversed (Yarrow et al., 2001; Morrone et al., 2005). This could explain poor and negative motion sensitivity as observed in the present study, although misperception of time seems to affect vision more generally, not motion exclusively and not only the dimension parallel to the saccade (Morrone et al., 2005). From a theoretical perspective, altered perceptions of time would make sense because they could help to cover up the discontinuities of vision caused by saccadic suppression (Yarrow et al., 2001). Thus, misperceptions of time and motion and their putative physiological correlates might reflect more than small imperfections of neural processing around the time of the saccade but functional processes of perceptual inference (von Helmholtz, 1910) that are designed to re-create an impression of visual continuity of a world that, *a priori*, is continuous most of the time. In this light, briefly appearing or disappearing stimuli as used here and in many other experimental paradigms could be regarded as “violations” of these *prior* assumptions (e.g., see Niemeier et al., 2003; for an explanation of the blanking effect; Deubel et al., 1996), and perceptual inference could explain why briefly flashed stimuli create falsely biased motion percepts (e.g., Castet et al., 2002).

To conclude, in the present study we revisit the question of how human motion perception changes around the time of saccadic eye movements. We find evidence for a mechanism of motion suppression that clearly starts before saccade onset and that, in and of itself, appears to be effective and specific in suppressing motion percepts of the blur caused by saccades. Furthermore, our data suggest that motion perception reverses at the center of saccades. We propose that this could be due to postsaccadic enhancement that might reverse responses in motion sensitive neurons. Our results are best explained by active mechanisms that are driven by extraretinal signals. Further research is required to explore the extent to which these processes can be modeled by principles of optimal inference.

## Conflict of Interest Statement

The authors declare that the research was conducted in the absence of any commercial or financial relationships that could be construed as a potential conflict of interest.
